# Health-related quality of life and physical activity level after a behavior change program at Norwegian healthy life centers: a 15-month follow-up

**DOI:** 10.1007/s11136-020-02554-x

**Published:** 2020-06-20

**Authors:** Ellen Eimhjellen Blom, Eivind Aadland, Guri Kaurstad Skrove, Ane Kristiansen Solbraa, Line Merethe Oldervoll

**Affiliations:** 1 Department of Sport, Food and Natural Sciences, Faculty of Education, Arts and Sports, Western Norway University of Applied Sciences, Campus Sogndal, Postbox 133, 6851 Sogndal, Norway; 2 Department of Public Health and Nursing, Faculty of Medicine and Health Sciences, Norwegian University of Science and Technology, 7491 Trondheim, Norway; 3 Department of Social Sciences, Møreforsking Molde AS, Britvegen 4, 6410 Molde, Norway; 4 LHL-Clinics Trondheim, Postbox 3015 Lade, 7441 Trondheim, Norway

**Keywords:** Physical activity, Accelerometer, Adults, Chronic health conditions, Primary health care, Behavior change program

## Abstract

**Purpose:**

The long-term impact of primary care behavior change programs on health-related quality of life (HRQoL) and physical activity (PA) level is unknown. The aim of this study was to investigate changes in HRQoL and PA among participants after a 3-month behavior change intervention at Norwegian healthy life center (HLCs) and at a 15-month follow-up. Furthermore, we aimed to study associations between changes in PA and HRQoL.

**Methods:**

We followed 524 adult participants (18–83 years), recruited from 32 HLCs in August 2016–January 2018, who provided data on HRQoL (SF-36) and PA (ActiGraph accelerometers) 12 months after a 3-month behavior change intervention. Changes in HRQoL and PA between baseline, 3-month and 15-month follow-ups, and associations between changes in PA and HRQoL were analyzed by linear mixed models.

**Results:**

All HRQoL dimensions improved from baseline to 3-month follow-up, and the improvements maintained at 15-month follow-up (mean 3.1–13.1 points, *p* < 0.001). PA increased from baseline to 3 months (mean 418 steps/day, *p* < 0.001), but declined from 3 to 15 months (mean − 371 steps/day, *p* < 0.001). We observed positive associations between changes in PA and HRQoL (0.84–3.23 points per 1000 steps/day, *p* < 0.023).

**Conclusions:**

Twelve months after completing a 3-month HLC intervention we found improved HRQoL, but not PA level. Still, there were positive associations between PA and HRQoL over this period, indicating that participants increasing their PA were more likely to improve their HRQoL.

## Introduction

Physical inactivity, together with smoking and an unhealthy diet, are among the most important behavioral risk factors for premature death and disability [1]. These risk factors tend to cluster and are more frequently observed among groups with low socioeconomic status and multiple chronic conditions [2–4]. Furthermore, individuals with multiple chronic conditions and low physical activity (PA) levels tend to report low health-related quality of life (HRQoL) [5–8]. Primary health care should therefore identify high-risk individuals to promote behavior change and HRQoL [9, 10].

Several programs targeting physically inactive high-risk individuals have been developed within the primary care, such as Exercise Referral Schemes in the UK and Physical Activity on Prescription in Sweden [11]. Previous systematic reviews have shown small to moderate short-term effects of such programs on participants’ PA level, physical fitness, obesity, and HRQoL [12–15]. However, only a limited number of studies have investigated the long-term impact of these programs, and objective measurements of PA are missing [12, 13, 16, 17].

In Norway, healthy life centers (HLCs), are implemented in about half of the municipalities as a primary health care service to promote beneficial PA-, diet- and tobacco behaviors [18]. Similar to other equivalent programs, the HLC program has shown mixed results concerning changes in PA, physical fitness, and HRQoL at both the short and long term [19–21]. However, previous studies included few HLCs, which limits the generalizability of their findings given great variability in how HLCs are adopted by the municipalities [18]. Moreover, large-scale studies investigating the long-term impact of the HLC program on participants’ PA level and HRQoL are lacking.

Although previous cross-sectional studies show positive associations between PA and HRQoL [5, 6, 22], results from longitudinal studies are conflicting [5, 6, 23]. In addition, subjective measures of PA are the foremost used method, which has limited precision [24], and hence might have led to attenuated relationships with HRQoL. Thus, there is a need for longitudinal studies using objective measures of PA [5, 6].

This study aimed to investigate changes in HRQoL and PA levels among participants attending an HLC behavior change program after a 3-month intervention and at a 15-month follow-up. Furthermore, we aimed to investigate associations between change in PA and HRQoL over this period.

## Methods

### Study design, setting, intervention and sample

We conducted a prospective observational study of participants attending the behavior change program carried out at HLCs in four Norwegian counties. Measurements were performed at baseline, following a 3-month intervention period, and 12 months after completion (15-month follow-up). Study setting, intervention components, and procedures are described in detail previously [25], and briefly explained in the following. Individuals were self-referred or referred from a general practitioner (GP), other health professionals, or the Norwegian Labor and Welfare Administration to the HLCs for support to promote PA, diet and/or smoking cessation. The HLC program offered individual consultations at the start and the end of the 3-month follow-up period, as well as additional consultations if needed. The consultations included personal goal setting and the design of a tailored plan aiming to change one or several behaviors. Interventions were based on salutogenic approaches [26], and delivered with motivational interview (MI) as the primary methodology [27]. Moreover, the HLC program consisted of group-based healthy eating courses (five times 2 h), smoking cessation courses (six to ten meetings), group meetings covering themes like clothing when exercising, and motivation, as well as supervised exercise groups at least twice a week. The exercise was mainly outdoor-based cardiorespiratory fitness- and strength training suited for persons with little or no previous exercise experience. Depending on the tailored plan, participants had access to one or several of these offers during the follow-up period, in addition to guidance on self-administered PA. Furthermore, the HLCs were cooperating with additional local providers of exercise in which the participants could engage with no or low cost, including non-governmental organizations and fitness centers. It was possible to prolong the first 3-month intervention period by one or several additional periods if needed to achieve behavior change [28]. Professions working at the HLCs were mainly physiotherapists and nurses [18]. Out of 60 HLCs established in the included four counties, 46 met the inclusion criteria described previously [25], and 32 accepted to take part in the study.

**Fig. 1:**
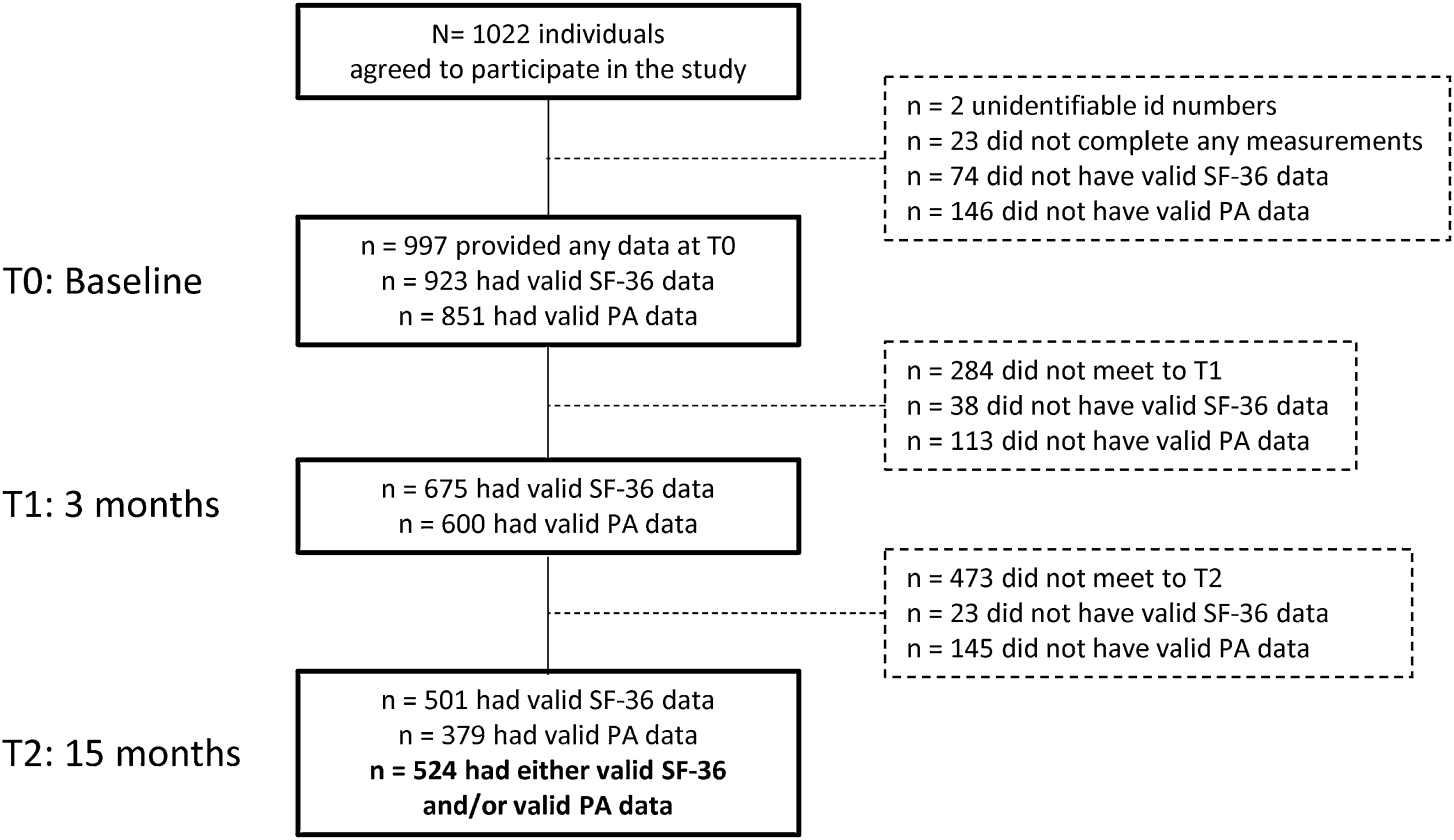
Flowchart of number of individuals included in the study. *PA* physical activity, *Valid PA* at least 4 valid days of PA assessment, *Valid SF-36* at least one dimension or health transition with ≥ 50% items completed

Individuals aged ≥ 18 years, meeting for their first consultation at one of the included HLCs in the period August 2016–January 2018, were invited to participate in the study. The only exclusion criterion was having been enrolled at an HLC program in the last 6 months. In total 1022 individuals agreed to participate in the study and provided written informed consent. A flowchart of the number of individuals included in the study, and the numbers completing valid PA- and HRQoL assessments at baseline (T0), 3 months (T1) and 15 months (T2) are presented in Fig. 1. The study was approved by the Regional Committee for Medical and Health Research Ethics (ref. 2016/546/REK midt), and has been carried out in accordance with the Declaration of Helsinki [29].

### Measurements

*Background characteristics* Sociodemographic variables (gender, age, nationality of origin, and educational status), chronic health conditions, smoking status and diet quality (eating at least 5 portions of fruit/berries/vegetables per day) was assessed by self-report. Whether participants met PA recommendations was assessed with ActiGraph accelerometers as described below. Occupational status was assessed through interviews carried out by HLC personnel. Height and body mass were measured by HLC personnel [25], and body mass index (BMI) calculated as kg/m^2^, and categorized as underweight (BMI < 20 kg/m^2^); normal weight (20–24.9 kg/m^2^); overweight (25–29.9 kg/m^2^); and obese (≥ 30 kg/m^2^) [30].

*Health-related quality of life* was measured by the Medical Outcome Short Form (MOS SF-36), version 1.0, translated and validated to the Norwegian population [31, 32]. SF-36 measures eight dimensions of quality of life through 35 items and one additional item measuring health transition (current perceived health compared to one year ago). The eight dimensions are physical functioning, role limitations due to physical health problems, bodily pain, general health, vitality, social functioning, role limitations due to emotional problems and mental health in general. The scores within all dimensions were calculated and transformed to a 0 (lowest health status)–100 (highest health status) scale, and missing items were handled according to the original manual if at least 50% of the items within the same dimension was competed [33]. Based on previous work on clinical populations, clinically important changes were considered at a ≥ 5-point difference [5, 34–37].

*Physical activity (PA) level* was measured by ActiGraph GT3X + accelerometers (ActiGraph, LLC, Pensacola, Florida, USA) sent by mail, with instructions of wearing the device on the right hip for seven consecutive days. Accelerometers were initialized using the ActiLife software (v 6.13.3, ActiGraph) with a sampling frequency of 30 Hz. The same software was used to download and process the PA data with a normal filtering option using 10-s epochs. Non-wear time was defined as a minimum of 60 consecutive min with zero counts, with allowance of up to 2 min of non-zero counts. Wear criteria for a valid measurement was set to at least 10 h of wear time per day for at least 4 days. Intensity-specific PA was calculated from the vertical axis using the Troiano cut-points [38] to determine sedentary time (min/day) as < 100 counts per min (cpm), light-intensity PA (LPA, min/day) as 100–2019 cpm, moderate-intensity PA (MPA, min/day) as 2020–5998 cpm, moderate-to-vigorous-intensity PA (MVPA, min/day) as ≥ 2020 counts per minute (cpm), and vigorous-intensity PA (VPA, min/day as ≥ 5999 cpm). We also reported overall PA (average cpm), the number of steps per day, and MVPA accumulated in 10 min bouts allowing for 2 min drop time (min/day). MVPA accumulated in bouts was used to determine the proportion of participants meeting the 150 min MVPA/week PA recommendation, defined as achieving an average of ≥ 21.4 min/day.

*Intervention characteristics* were assessed by retrospective interviews and reported by HLC personnel: Referral institution, primary reasons for referral (PA, diet or smoking cessation, with possibilities of reporting more than one primary reason), and number of intervention periods after the first 3-month intervention.

### Statistics

Descriptive continuous data are presented as means (standard deviation, SD) and categorical data as frequencies (numbers, n), or medians (interquartile range, IR). Differences between completers (completing either valid SF-36 and/or PA measurement(s) at T2 (*n* = 524) and drop-outs (*n* = 473), were analyzed by using a linear mixed model (LMM) for continuous or ordinal outcome variables, or generalized estimating equations (GEE) for dichotomous outcome variables [39]. Changes between the three time points (T0–T1, T1–T2, and T0–T2) were analyzed using an LMM. LMM handles missing data using maximum likelihood estimation and by using all available data for each individual at each test time point [40, 41]. We determined the main effect of time for each outcome using separate models, including random intercepts of subject and HLC. Within models fitted with PA outcomes, we additionally adjusted for season and wear time. We also added whether participants completed one or more intervention periods to this model to determine whether multiple periods influenced changes over time. Change over time (Δ) is presented as unstandardized regression coefficients (B), with 95% confidence intervals (CIs). The clustering effects of subjects and HLCs are presented as Intraclass correlation coefficients (ICCs). Associations between changes in PA variables (continuous, independent variables) and changes in HRQoL dimensions (continuous, dependent variables) were investigated by associating change scores (Δ T0–T1, Δ T1–T2 and Δ T0–T2) in separate LMM models. HLC was included as a random intercept, and we adjusted for change in wear time and value from the first test time point (T0 or T1 as appropriate) of the outcome in each model. Associations are presented as unstandardized regression coefficients (B), 95% CIs, and statistical significance levels (*p*-values ≤ 0.05 indicated statistically significant findings). All analyses were performed using IBM Statistics for Windows, Version 26.0 (SPSS Inc., Chicago, Illinois, USA).

## Results

### Participant characteristics

A total of 524 participants (51% of individuals included at baseline) completed either valid SF-36 and/or valid PA measurements at the 15-month follow-up and were included in the analysis (Fig. 1). Participants were 18–83 years of age, mostly women (70%) with Norwegian origin (92%) and had a median of two chronic conditions (IR 2) (Table 1).

**Table 1:** Baseline characteristics of the Norwegian healthy life centers’ study sample (2016**–**2018), *n* = 524

Variables	% (*n*)
Age (years) (SD)	52.7 (13.8)
Educational level	
Primary school, 0–10 years	16.6 (86)
High school, 11–13 years	47.8 (248)
College/university, ≤ 3 years	20.2 (105)
College/university, > 3 years	15.4 (80)
Occupational status^a^	
Working^b^	41.8 (218)
Sick-leave^b^	15.4 (80)
Social-benefits^b^	38.8 (202)
Retired	19.2 (100)
Student	0.8 (4)
Other	4.2 (22)
Health status	
BMI (kg/m^2^) (SD)	32.3 (7.0)
Underweight	0.6 (3)
Normal weight	12.7 (64)
Overweight	25.9 (130)
Obese	60.8 (305)
Chronic conditions^a^	
No disease	10.1 (52)
NCD risk factors	59.6 (310)
Musculoskeletal disorders	42.0 (217)
NCDs	31.5 (164)
Mental disease	24.8 (128)
Other diseases	13.2 (68)
Smoking	20.0 (103)
Not meeting diet recommendation^c^	75.4 (386)
Not meeting PA recommendations^d^	80.0 (419)
Sum of risk behaviors (PA, diet and/or smoking)	
0	5.3 (27)
1	27.9 (141)
2	52.8 (267)
3	14.0 (71)
Referral institution	
GP	55.2 (285)
Others	22.9 (118)
Self-referred	17.4 (90)
Labor and welfare administration	4.5 (23)
Primary behavior to change^a^	
PA	90.8 (473)
Diet	34.4 (178)
Tobacco	2.7 (14)

*SD* standard deviation, *BMI* Body Mass Index, *NCD* non-communicable disease, *PA* physical activity, *GP* general practitioner

^a^Possible to report more than one status/condition/behavior

^b^Full- or part-time

^c^Five portions of fruits or vegetables per day on average

^d^150 min moderate- and vigorous PA/week

While 53% of the participants received one intervention period, respectively, 23%, 10%, 6%, and 3% received 2, 3, 4, and 5 intervention periods over the 15 months, whereas 5% did not report their number of additional periods.

Participants completing the 15-month follow-up (*n* = 524) did not differ from the drop-outs (*n* = 473) regarding gender (*p* = 0.151), BMI (*p* = 0.665) or number of chronic conditions (*p* = 0.373). However, participants completing the 15-month follow-up were older (*p* < 0.001) and were more likely to be retired (*p* < 0.001), to be of Norwegian origin (*p* = 0.001), to have higher education (*p* = 0.004), to have non-communicable diseases (NCDs) (*p* = 0.042), and to be a non-smoker (*p* < 0.001), whereas they were less likely to be a student (*p* = 0.032), to be on sick-leave (*p* = 0.035), or to have a mental disease (*p* = 0.003) compared to drop-outs.

### Change in health-related quality of life

HRQoL improved statistically significantly from baseline to the 3-month follow-up within all dimensions, ranging from a mean of 3.1 points increase in bodily pain to 9.9 points increase in role physical (Table 2). The improvement was maintained for all dimensions, except for mental health showing a further improvement from 3- to 15-month follow-up. The changes observed at 15 months were above a 5-point difference within all dimensions except for physical functioning and bodily pain. While health transition increased from baseline to 3-month follow-up, it declined between 3- and 15-month follow-ups but showed an overall increase from baseline to 15-month follow-up (Table 2). Changes were similar for participants attending only one 3-month intervention period and participants attending multiple intervention periods (all *p* > 0.076).Change in physical activity.

**Table 2:** Change in Health-related quality of life (HRQoL) scores (0–100) from baseline to 15-month follow-up among Norwegian HLC participants (*n* = 501)

	Mean (SD) scores			Change scores over time B (95% CI), *p*						ICC	
HRQoL	Baseline (T0)	3 months (T1)	15 months (T2)	T0–T1	*p*	T1–T2	*p*	T0–T2	*p*	Subject	HLC
Dimensions											
Physical functioning	74.9 (17.8)	78.0 (19.7)	78.1 (19.9)	3.3 (1.9, 4.6)	< 0.001	0.2 (− 1.5, 1.2)	0.796	3.1 (1.8, 4.5)	< 0.001	0.66	0.02
Role physical	49.5 (42.0)	58.6 (43.3)	62.3 (42.1)	9.9 (6.4, 13.4)	< 0.001	3.2 (− 0.4, 6.7)	0.082	13.1 (9.6, 16.6)	< 0.001	0.54	0.02
Bodily pain	54.6 (27.7)	57.3 (27.8)	58.5 (27.7)	3.1 (1.0, 5.2)	0.004	0.9 (− 1.2, 3.1)	0.391	4.0 (1.9, 6.1)	< 0.001	0.63	0.00
General health	53.9 (22.5)	59.5 (22.8)	59.8 (22.2)	5.8 (4.3, 7.4)	< 0.001	0.4 (− 1.1, 2.0)	0.582	6.3 (4.7, 7.8)	< 0.001	0.66	0.03
Vitality	42.4 (20.5)	47.5 (20.9)	47.9 (20.5)	5.3 (3.6, 7.0)	< 0.001	0.4 (− 1.3, 2.1)	0.626	5.7 (4.1, 7.4)	< 0.001	0.55	0.02
Social functioning	69.9 (29.1)	76.4 (25.5)	76.6 (26.9)	6.5 (4.4, 8.6)	< 0.001	0.3 (− 1.8, 2.5)	0.777	6.8 (4.7, 8.6)	< 0.001	0.57	0.03
Role emotional	64.8 (42.3)	73.8 (38.4)	76.1 (37.2)	8.7 (4.9, 12.5)	< 0.001	2.4 (− 1.4, 6.2)	0.219	11.1 (7.3, 14.9)	< 0.001	0.35	0.04
Mental health	69.3 (20.3)	73.0 (18.5)	74.1 (18.6)	3.6 (2.2, 5.0)	< 0.001	1.5 (0.1, 2.9)	0.038	5.1 (3.7, 6.4)	< 0.001	0.63	0.03
Health transition	51.0 (39.6)	69.8 (26.0)	66.3 (26.8)	19.5 (16.5, 22.6)	< 0.001	− 4.0 (− 7.1, − 0.9)	0.011	15.5 (12.5, 18.5)	< 0.001	0.19	0.01

Mean (SD) scores derived from descriptive statistics. Change scores over time (B, 95% CI, *p*, and ICC) derived from linear mixed models accounting for random intercepts of subject and HLC. Clinically important changes are considered at a ≥ 5-point difference

*HLC* healthy life center, *HRQoL* health-related quality of life, *SD* standard deviation, *CI* confidence interval, *ICC* intraclass correlation coefficient, *Test time points*: *T0* Baseline, *T1* 3 months, *T2* 15 months

While PA levels generally increased, and SED decreased, from baseline to 3 months, PA levels decreased, and SED increased, from 3 to 15 months (Table 3). From baseline to 15-month follow-up there were no statistically significant changes in PA, except for a decline in time spent in 10 min bouts of MVPA. Changes were similar for participants attending one or multiple intervention periods (all *p* > 0.192).

**Table 3:** Change in physical activity (PA) levels from baseline to 15-month follow-up among Norwegian HLC participants (*n* = 379)

	Mean (SD) values			Change over time B (95% CI), *p*						ICC	
PA outcomes	Baseline (T0)	3 months (T1)	15 months (T2)	T0–T1	*p*	T1–T2	*p*	T0–T2	*p*	Subject	HLC
SED (min/day)	618 (70)	609 (76)	616 (73)	− 6.2 (− 11.2, − 1.2)	0.016	6.4 (1.9, 10.8)	0.005	0.2 (− 4.8, 5.1)	0.950	0.655	0.053
LPA (min/day)	177 (50)	183 (53)	181 (56)	4.1 (− 0.2, 8.3)	0.060	− 2.4 (− 6.2, 1.4)	0.215	1.7 (− 2.5, 5.9)	0.428	0.588	0.115
MPA (min/day)	35.4 (21.6)	38.0 (23.5)	34.2 (22.6)	2.1 (0.1, 4.0)	0.043	− 3.9 (− 5.7, − 2.1)	< 0.001	− 1.9 (− 4.0, 0.1)	0.061	0.665	0.018
MVPA (min/day)	36.2 (22.3)	39.0 (24.4)	35.1 (23.5)	2.0 (− 0.0, 4.1)	0.051	− 4.0 (− 5.8, − 2.2)	< 0.001	− 2.0 (− 4.0, 0.1)	0.057	0.667	0.016
VPA (min/day)	0.9 (2.7)	1.0 (2.1)	0.9 (2.2)	− 0.0 (− 0.3, 0.2)	0.866	− 0.1 (− 0.3, 0.1)	0.483	− 0.1 (− 0.4, 0.1)	0.424	0.550	0.000
MVPA bouts (min/day)	11.4 (15.4)	12.5 (15.0)	9.7 (15.8)	0.8 (− 0.8, 2.4)	0.334	− 2.7 (− 4.1, − 1.3)	< 0.001	− 1.9 (− 0,3, − 3.5)	0.018	0.522	0.071
Overall PA (cpm)	283 (117)	301 (130)	282 (127)	12.5 (1.6, 23.4)	0.025	− 19.4 (− 29.0, − 9.7)	< 0.001	− 6.9 (− 17.7, 3.9)	0.210	0.693	0.003
Steps (number/day)	6121 (2569)	6618 (2785)	6266 (2809)	418 (199, 636)	< 0.001	− 371 (− 565, − 178)	< 0.001	47 (− 170, 263)	0.672	0.704	0.015

Mean (SD) values derived from descriptive statistics. Change scores over time (B, 95% CI, *p*, and ICC) derived from linear mixed models adjusted for wear time and season, taking into account random intercepts of subject and HLC

*HLC* healthy life center, *PA* physical activity, *SED* sedentary time, *LPA* light PA, *MPA* moderate PA, *MVPA* moderate and vigorous PA, *VPA* vigorous PA, *cpm* counts per minute, *SD* standard deviation, *CI* confidence interval, *ICC* intraclass correlation coefficient, *Test time points*: *T0* Baseline, *T1* 3 months, *T2* 15 months

### Associations between change in physical activity and change in health-related quality of life

Overall, changes in PA were positively associated, and SED negatively, with changes in all HRQoL dimensions, except for role emotional (Table 4).

**Table 4:** Associations (B (95% CI)) between changes in MVPA, LPA, SED and Steps and changes in HRQoL dimensions’ scores (0–100 scale) among Norwegian HLC participants (*n* = 379)

	Physical functioning	Role physical	Bodily pain	General health	Vitality	Social functioning	Role emotional	Mental health
MVPA (min/day)								
T0–T1	0.11 (0.04, 0.17)*	0.24 (0.06, 0.43)*	0.15 (0.04, 0.26)*	0.03 (− 0.06, 0.11)	0.09 (− 0.01, 0.18)	0.12 (0.01, 0.24)*	0.11 (− 0.07, 0.29)	0.05 (− 0.02, 0.13)
T1–T2	0.13 (0.05, 0.22)*	0.23 (0.01, 0.44)*	0.10 (− 0.03, 0.23)	0.12 (0.03, 0.21)*	0.12 (0.02, 0.22)*	0.12 (− 0.01, 0.24)	− 0.01 (− 0.21, 0.20)	0.02 (− 0.06, 0.10)
T0–T2	0.14 (0.06, 0.23)*	0.13 (− 0.07, 0.33)	0.11 (− 0.01, 0.22)	0.09 (0.00, 0.17)	0.13 (0.04, 0.22)*	0.14 (0.02, 0.26)*	0.06 (− 0.12, 0.24)	0.04 (− 0.04, 0.12)
LPA (min/day)								
T0–T1	0.02 (− 0.01, 0.06)	0.02 (− 0.08, 0.12)	0.02 (− 0.03, 0.08)	0.01 (− 0.04, 0.05)	0.04 (− 0.01, 0.09)	− 0.01 (− 0.06, 0.05)	0.05 (− 0.04, 0.15)	0.03 (− 0.01, 0.07)
T1–T2	0.08 (0.03, 0.12)*	0.01 (− 0.10, 0.11)	− 0.01 (− 0.08, 0.05)	0.04 (0.00, 0.08)	0.04 (− 0.01, 0.08)	0.01 (− 0.04, 0.07)	− 0.01 (− 0.10, 0.09)	0.04 (0.00, 0.09)*
T0–T2	0.04 (0.00, 0.08)	0.06 (− 0.04, 0.16)	0.03 (− 0.03, 0.09)	0.03 (− 0.01, 0.07)	0.06 (0.01, 0.10)*	0.06 (0.00, 0.12)*	0.09 (0.00, 0.18)	0.03 (0.00, 0.07)
SED (min/day)								
T0–T1	− 0.03 (− 0.06, 0.00)*	− 0.06 (− 0.14, 0.02)	− 0.04 (− 0.09, 0.00)	− 0.01 (− 0.05, 0.03)	− 0.04 (− 0.08, 0.00)*	− 0.02 (− 0.07, 0.03)	− 0.06 (− 0.13, 0.02)	− 0.03 (− 0.06, 0.00)
T1–T2	− 0.08 (− 0.11, − 0.04)*	− 0.04 (− 0.13, 0.05)	− 0.01 (− 0.06, 0.05)	− 0.05 (− 0.08, − 0.01)*	− 0.05 (− 0.09, − 0.01)*	− 0.03 (− 0.08, 0.02)	0.01 (− 0.08, 0.09)	− 0.03 (− 0.06, 0.00)
T0–T2	− 0.05 (− 0.09, − 0.02)*	− 0.06 (− 0.15, 0.02)	− 0.04 (− 0.09, 0.01)	− 0.04 (− 0.07, 0.00)*	− 0.06 (− 0.10, − 0.03)*	− 0.07 (− 0.12, − 0.02)*	− 0.07 (− 0.15, 0.01)	− 0.04 (− 0.07, 0.00)*
1000 Steps/day								
T0–T1	0.96 (0.31, 1.60)*	2.56 (0.81, 4.32)*	1.46 (0.41, 2.51)*	0.66 (− 0.14, 1.46)	1.17 (0.30, 2.03)*	1.57 (0.51, 2.63)*	1.42 (− 0.27, 3.11)	0.84 (0.14, 1.54)*
T1–T2	2.15 (1.31, 2.99)*	3.23 (1.11, 5.35)*	1.50 (0.23, 2.76)*	1.61 (0.72, 2.49)*	1.63 (0.67, 2.59)*	1.41 (0.20, 2.62)*	0.90 (− 1.13, 2.94)	0.13 (− 0.67, 0.94)
T0–T2	2.02 (1.26, 2.79)*	1.67 (− 0.15, 3.50)	1.28 (0.20, 2.35)*	1.02 (0.23, 1.81)*	1.56 (0.74, 2.37)*	1.55 (0.45, 2.65)*	1.62 (− 0.03, 3.27)	0.54 (− 0.19, 1.28)

Associations are presented as unstandardized regression coefficients (B) with associated 95% confidence intervals (CI) derived from linear mixed models including change-values of PA and HRQoL between two test time points, adjusted for change in accelerometer wear time and outcome value from the first test point within the model, taking random intercept of the subject into account

*MVPA* moderate-to-vigorous-intensity physical activity (PA), *LPA* light -intensity PA, *SED* sedentary time, *HRQoL* health-related quality of life, *HLC* healthy life centers, *Test time points*: *T0* Baseline, *T1* 3 months, *T2* 15 months

*Statistically significant associations at *p* < 0.05

Regarding intensity-specific PA, associations were strongest and most consistent across HRQoL dimensions for MVPA. While a 1 min/day increased level of MVPA was associated with improvements of 0.11–0.24 points HRQoL (physical functioning, role physical, bodily pain, general health, vitality, and social functioning), the same amount of LPA (positively) and SED (negatively) was associated with changes of 0.03–0.08 points HRQoL (physical functioning, general health (only SED), vitality, social functioning, and mental health).

Furthermore, an increase of 1000 steps/day was associated with an improvement of 0.84–3.23 points HRQoL (all dimensions except for role emotional) (Table 4).

## Discussion

The present study showed that HRQoL was improved after participation at a 3-month HLC behavior change program within the primary care. Changes for several HRQoL dimensions are regarded as clinically relevant, and the immediate improvements were maintained 12 months later. Although we found no change in PA level over the long term, changes in PA and HRQoL over the intervention period and the long-term follow-up were positively associated. These findings indicate that participants increasing their PA levels were more likely to improve their HRQoL.

Our results demonstrating small initial improvements in PA levels immediately after the behavior change intervention, however, not maintained in the long term, are in line with previous studies of such programs within the primary care [12, 13, 15]. Thus, our results derived from accelerometry, confirm previous findings derived from self-report instruments and strengthen previous research indicating that behavior change programs within primary care have limited long-term impact on participant’s PA level.

However, the participants’ HRQoL improved statistically significantly across all the eight measured HRQoL dimensions, as well as in health transition, following the 3-month intervention. All improvements were maintained at the 15-month follow-up, with even additional improvements in mental health. For all dimensions except for physical functioning and bodily pain, the long-term changes were above a 5-point difference, which has been considered clinically important [5, 34–36]. Previous evidence on primary care PA programs’ impact on HRQoL is mixed. Although some studies have found positive impact [12, 16, 20, 37, 42–44], our findings are in conflict with other studies showing minimal effects [19, 45–48].

A major challenge when comparing results between studies of behavior change programs within primary care is the extensive variety of intervention components between countries, and even within countries [11, 49]. For example, the Swedish Physical Activity on Prescription model is mainly based on behavior consultations by GPs, or other health professionals within primary care, and a prescription to self-administered PA [13], whereas Exercise Referral Schemes in the UK mainly refer users to a third-party provider of exercise outside primary care [11]. In the Norwegian HLC model, behavior change courses and consultations regarding diet and smoking cessation, in addition to PA, are organized both within the primary care and also in cooperation with other providers, in addition to encouraging self-administered exercise [28]. The municipalities in Norway have furthermore adapted the HLC model differently according to local competence and resources available [18]. These variations may explain some of the inconsistent findings among studies, and further investigation is needed to identify which specific program features that are the most favorable for long-term success in improving participant’s PA and HRQoL.

The HLC population report multiple chronic conditions and low HRQoL at baseline compared to the general population [22]. Furthermore, they report low self-efficacy and great psychological barriers to behavior change acquired from past life experiences [50, 51]. Hence, their perceived change in quality of life is an important outcome of a health intervention [52]. However, our finding that HRQoL improved over time, whereas PA did not, question the importance of PA for quality of life. Despite any covariation of these measures on a group level over the long-term, our weak positive associations between PA and HRQoL, suggest that relationship exist on an individual level. However, due to the observational design, we cannot draw any conclusion with regard to causality.

The finding of positive associations between change in PA and change in HRQoL confirms our previous cross-sectional analysis of the relationship between PA and HRQoL within this population [22]. The longitudinal analyses presented herein revealed additional associations with MVPA for several dimensions which were non-significant in the cross-sectional study. These results are interesting since previous cohorts have found weaker associations between MVPA or leisure-time PA and HRQoL in longitudinal analyses than in cross-sectional analyses [23, 53]. The magnitude of associations observed in the current study ranged from 0.11 to 0.24 points improved SF-36 points per increased min/day of MVPA, corresponding to an increase of 1.1 to 2.4 points for every 10 min/day increase in MVPA (or about 1 h/week). Although this magnitude of association is relatively weak, it is larger than observed by previous studies (0.09 to 0.39 points increase in SF-36 points for every 1 h/week increase of MVPA or leisure-time PA) [23, 53]. Furthermore, we observed that an increase in PA corresponding to about 2000 steps per day was associated with more than 5 points improvement in HRQoL, which is considered as a clinically important change.

Although we cannot conclude with respect to the cause of the divergence in these studies’ results, it is well-known that self-report methods to assess PA, as applied in the previous studies, have important limitations compared to objective PA assessment by accelerometry, as applied herein [23, 53]. Importantly, in two previous systematic reviews investigating associations between PA and HRQoL in adults, there was only one longitudinal study using accelerometry [5, 6]. Subjective assessments of PA are known to be limited by certain biases such as recall- and social desirability biases, and have limitations in measuring intensity-specific and overall PA level precisely [54]. These measurement errors cause regression dilution bias and thus attenuated associations with health. Hence, the current study’s findings extend the previous knowledge about associations between PA and HRQoL within high-risk adults.

The main strengths of the present study are the large sample included, the long follow-up time, and the objective measurement of PA. Despite accelerometers’ limitations in measuring certain types of PA, such as upper-body movement, cycling, and water-activities, accelerometry is superior to the use of subjective measurement methods [24, 55].

The lack of a true experimental design and a control group excludes the possibility of drawing causal conclusions. Moreover, the relatively high drop-out of individuals with certain characteristics limits the ability to generalize the findings to groups with mental disorders, individuals being on sick-leave, younger individuals, and individuals with non-Norwegian origin. Moreover, those completing the long-term follow-up were likely individuals achieving more favorable results than those not providing data. Hence, the favorable changes observed might be over-estimated. However, the proportion of drop-out in the present study is comparable to previous observational studies of equivalent programs [20, 43]. Finally, we did not correct for multiple comparisons. However, emphasis is placed on clinical rather than statistical significance in interpretation of results.

Our study showed no long-term changes in HLC participants’ PA levels. Although previous controlled clinical trials of PA interventions have shown positive effects among healthy populations [56] and groups with specific conditions [57, 58], implementing such programs into a real-life setting is challenging, and the knowledge about effective interventions within primary care to achieve long-term effects among high-risk groups remains unclear [12, 59]. The HLC population comprises a heterogeneous group with multiple health challenges [22, 50, 51, 60]. Although group-based interventions enhancing social support have been found effective to promote PA [61], tailoring group-based programs to suit all groups’ requirements is demanding [49]. Given the substantial psychological challenges among participants attending the HLC program [50, 51], the staffs’ competence on how to promote socio-psychological health is of particular importance [62]. Furthermore, extensive follow-up has been found more beneficial than less comprehensive interventions to achieve long-term behavior changes within high-risk groups [49, 59, 63, 64]. Thus, we suggest that future studies should investigate the impact of the staffs’ expertise on socio-psychological support and a comprehensive follow-up on HLC participants’ PA level over the long term.

## Conclusion

Participants at the Norwegian HLC behavior change program improved their HRQoL substantially over 15 months, although their PA level did not change. These results indicate that the program has an immediate positive impact on participants’ quality of life that is maintained 12 months later. Furthermore, although the HLC program did not have a long-term impact on the participants’ PA level, changes in PA were positively associated with changes in HRQoL. Thus, participants improving their PA level were more likely to improve their HRQoL. Hence, developing more effective intervention components to promote long-term changes in PA among high-risk groups could be beneficial to also promote HRQoL within this population. However, as both the HLC population and the HLC settings are highly heterogeneous, further studies are needed to investigate both individual and organizational predictors of success, to further develop effective behavior change programs targeting high-risk adults.

## Data Availability

Anonymous dataset is available from corresponding author upon request.
